# A Comparison of Urine Dilution Ability between Adult Dominant Polycystic Kidney Disease, Other Chronic Kidney Diseases, and Healthy Control Subjects: A Case-Control Study

**DOI:** 10.1155/2020/4108418

**Published:** 2020-12-02

**Authors:** M. H. Malmberg, F. H. Mose, E. B. Pedersen, J. N. Bech

**Affiliations:** University Clinic in Nephrology and Hypertension, Regional Hospital Jutland West, University of Aarhus, Aarhus, Denmark

## Abstract

The final dilution of urine is regulated via aquaporin-2 water channels in the distal part of the nephron. It is unclear whether urine dilution ability in autosomal dominant polycystic kidney disease patients (ADPKD patients) differs from other patients with similar degree of impaired renal function (non-ADPKD patients). The purpose of this case control study was to measure urine dilution ability in ADPKD patients compared to non-ADPKD patients and healthy controls. *Methods*. Eighteen ADPKD, 16 non-ADPKD patients (both with chronic kidney disease, stage I-IV), and 18 healthy controls received an oral water load of 20 ml/kg body weight. Urine was collected in 7 consecutive periods. We measured free water clearance (C_H2O_), urine osmolality, urine output, fractional excretion of sodium, urine aquaporin2 (u-AQP2), and urine epithelial sodium channel (u-ENaC). Blood samples were drawn four times (at baseline, 2 h, 4 h, and 6 hours after the water load) for analyses of plasma osmolality, vasopressin, renin, angiotensin II, and aldosterone. Brachial and central blood pressure was measured regularly during the test. *Results*. The three groups were age and gender matched, and the patient groups had similar renal function. One hour after water load, the ADPKD patients had an increased C_H2O_ compared to non-ADPKD patients (2.97 ± 2.42 ml/min in ADPKD patients vs. 1.31 ± 1.50 ml/min in non-ADPKD patients, *p*0.029). The reduction in u-AQP2 and u-ENaC occurred earlier in ADPKD than in non-ADPKD patients. Plasma concentrations of vasopressin, renin, angiotensin II, and aldosterone and blood pressure measurements did not show any differences that could explain the deviation in urine dilution capacity between the patient groups. *Conclusions*. ADPKD patients had a higher C_H2O_ than non-ADPKD patients after an oral water load, and u-AQP2 and u-ENaC were more rapidly reduced than in non-ADPKD patients. Thus, urine-diluting capacity may be better preserved in ADPKD patients than in non-ADPKD patients.

## 1. Introduction

The final dilution of the urine is regulated via vasopressin dependent aquaporin-2 water channels in the apical membrane of kidney principal cells [1–3]. The ability to dilute the urine can be measured by a urine diluting test, in which the participants undergo water loading [4]. Urine dilution is generally reduced in kidney diseases, but the severity and underlying mechanisms may deviate in different kidney disease [5].

Autosomal dominant polycystic kidney disease (ADPKD) is a common genetic disease with multiple cysts in the kidneys, leading to decline in kidney function [6]. Previous studies have shown that vasopressin stimulates the growth of kidney cysts and may be a significant factor in the pathogenesis and progression of ADPKD [7–10]. However, it is unclear whether urine dilution ability in ADPKD differs from other patients with similar degree of impaired renal function (non-ADPKD patients).

The purpose of the present case-control study was to perform a urine dilution test in patients with ADPKD (Group1), in patients with chronic kidney diseases without ADPKD (Group 2) and in healthy control subjects (Group 3). The three groups have previously participated in a water deprivation test on another examination day, at least 10 days apart. The results have been published previously [11].

The mechanisms differ for development of decline in renal function in ADPKD from other kidney diseases. Thus, we hypothesized that ADPKD patients have a reduced urinary dilution ability compared to other patients with a similar degree of impaired kidney function (non-ADPKD patients) and compared to healthy controls. We also hypothesized that the difference in urine diluting ability between patients with chronic renal disease and healthy controls could be partly explained by change, in the vasoactive hormone systems or blood pressure.

## 2. Materials and Methods

### 2.1. Study Design

The design was as a randomized, case-control study with three groups. Group 1 comprised patients with ADPKD, Group 2 comprised patients with chronic kidney diseases other than ADPKD (non-ADPKD), and Group 3 comprised healthy control subjects. The subjects participated in two different examination days, at least 10 days apart. A water deprivation test on one day and a water loading test on the other were performed.

### 2.2. Recruitment

Participants were recruited in the period between September 2017 and November 2018. In Groups 1 and 2, patients were recruited from the Outpatient Clinic of Nephrology in University Clinic in Nephrology and Hypertension, Department of Medicine at Holstebro Hospital, Denmark. Healthy controls in Group 3 were recruited by advertising in the local newspaper. All three groups were matched regarding age and gender. The two patient groups were matched regarding eGFR, calculated by CKD-EPI formula.

### 2.3. Randomization

The website *redcap.au.dk* randomized the order in which the subjects participated in each examination day with altering block sizes.

### 2.4. Subjects

#### 2.4.1. Inclusion Criteria

*Group 1*. Age 18 years or older: men and unfertile women or fertile women using safe contraception throughout the trial period (safe contraception is defined as birth control pills, spiral, depot injection of progestogen, subdermal implantation, hormonal vaginal ring, transdermal patch, sexual abstinence, or sterilization). Kidney function corresponds to chronic kidney disease (CKD) stages I-IV (eGFR >15 mL/min/1.73 m^2^). ADPKD was diagnosed by genetic testing for PKD1 and PKD2 mutations or presence of one of the following ultra-sonographic findings in accordance with the classic Ravine criteria. Thus, patients were included with a negative family history of ADPKD, when they had more than 10 cysts in each kidney without other causes of extrarenal or renal cyst formations. In addition, patients with a family history of ADPKD were included depending on age and number of cysts. The demand for inclusion was ≥3 cysts uni- or bilaterally in age 18–39 years, ≥2 cysts in each kidney in age 40–59 years, and ≥4 cysts in age higher than 60 years [12]. *Group 2*. Same criteria as for Group 1 but without the criteria for ADPKD. *Group 3*. Age 18 years or older. Healthy men and unfertile women or fertile women using safe contraception throughout the trial period (as indicated for Group 1).

#### 2.4.2. Exclusion Criteria

*Groups 1 and 2*. Previous kidney transplantation or kidney operation, diabetes mellitus, lithium nephropathy, medullary cystic kidney disease, neoplastic disease, pregnancy or breastfeeding, withdrawn consent, intolerance to or unacceptable side effects to water loading test, alcohol or drug abuse, or blood pressure >170/110 mm Hg despite treatment with metoprolol and/or amlodipine. *Group 3*. Arterial hypertension, i.e., office blood pressure above 140 mm Hg systolic and/or 90 mm Hg diastolic, significant clinical signs of heart disease, diseases of the lungs, liver, kidneys, endocrine organs, brain or neoplastic disorders, alcohol or drug abuse, medical treatment except oral contraceptives, smoking, pregnancy, or breastfeeding, clinically significant abnormal findings in blood tests (plasma concentration of sodium, potassium, albumin, creatinine, bilirubin, alanine-amino-transaminase (ALAT), alkaline phosphatase, cholesterol, calcium, phosphate, parathyroid hormone (PTH), thyroid-stimulating hormone (TSH), blood concentration of hemoglobin, leukocytes, platelets, and glycosylated hemoglobin A1c (HbA1c), or in the urine sample (leukocytes, nitrite, blood, glucose, and albumin), clinically significant changes in electrocardiogram, blood donation within the last month before the test date in the first trial sequence, and intolerance to or unacceptable side effects to water loading test.

#### 2.4.3. Withdrawal Criteria

Development of exclusion criteria and serious or unacceptable side effects are the withdrawal criteria.

### 2.5. Effect Variables

The primary effect variable was free water clearance (C_H20_). The secondary effect variables were (1) urine osmolality (u-Osm), urine output (UO), and fractional excretion of sodium (FE_Na_); (2) urine excretion of aquaporin 2 water channels (u-AQP2) and urine excretion of a fraction of the epithelial sodium channels (u-ENaC); (3) plasma concentrations of vasopressin (p-AVP), renin (PRC), angiotensin II (p-AngII) and aldosterone (p-Aldo), and plasma osmolality (p-Osm); (4) brachial and central blood pressure (bBP and cBP), augmentation index (AIx), and pulse wave velocity (PWV).

### 2.6. Number of Subjects

With a minimal relevant difference of 0.7 ml/min in C_H2O_ with an estimated standard deviation (SD) of 0.9 ml/min, 16 subjects were needed using a level of significance of 5% and a statistical power of 90%. To counteract any drop out, 20 patients with ADPKD, 20 non-ADPKD patients, and 20 healthy subjects were included.

### 2.7. Antihypertensive Medications

Antihypertensive medications including diuretics, angiotensin-converting enzyme inhibitors, and angiotensin-II inhibitors were discontinued or substituted with metoprolol 25 mg and/or amlodipine 5 mg, one or two times a day 14 days prior to each test day. During the study period, bBP was monitored using a home blood pressure monitor. At a blood pressure of >170/110 mm Hg, metoprolol 25 mg and/or amlodipine 5 mg was given and increased up to metoprolol 100 mg and/or amlodipine 10 mg. Subjects were discontinued from the study if blood pressure above 170/110 mm Hg continued despite treatment with metoprolol tartrate or metoprolol succinate 100 mg and/or amlodipine 10 mg. The usual antihypertensive treatment was resumed immediately after the examination day ended.

### 2.8. Ethics

The Regional Committee on Health Research Ethics approved the study (case number: 1-10-72-147-17). The study was done in agreement with the Declaration of Helsinki and was registered at clinicaltrials.gov (identifier: **NCT04363554**). Written informed consent was obtained from each subject.

## 3. Experimental Procedure

The study took place at the Laboratory in the University Clinic in Nephrology and Hypertension, Regional Hospital Jutland West. On the day prior to the examination day, all subjects consumed their habitual intake of food and beverage but no alcohol.

A 24-hour urine sample was collected before the participants arrived at the University Clinic in Nephrology and Hypertension from 07.30 AM the day before examination to 07.30 AM on the examination day (Period 1). Thereafter, urine was collected in six consecutive periods (Periods 2–7) of each one-hour's duration from 07.30 AM to 01.30 PM. After Period 2, the subjects received an oral water load of 20 ml/kg body weight during 20 minutes. Blood samples were drawn after each period. Urine samples were collected by voiding in standing or sitting position after blood samples had been collected. Patients were kept in a supine position in a quiet and temperature-controlled room (22–25°C). BBP and cBP were measured every 20 minutes by Mobil-O-Graph® PWA on one of the subject's upper arm. In the other arm, an intravenous catheter was placed to collect blood samples. In urine, we measured UO, u-Osm, u-Na (urine sodium), u-Cr (urine creatinine), u-AQP2, u-ENaC, and u-Alb (urine albumin) in all samples. In plasma, we measured p-AVP, PRC, p-AngII, and p-Aldo after periods 2, 4, 6, and 7. After all periods, we measured p-Osm, p-Na (plasma sodium), p-Cr (plasma creatinine), and p-Alb (plasma albumin).

### 3.1. Measurements

#### 3.1.1. Renal Function

Estimated GFR (eGFR) was calculated using the CKD-EPI formula. Clearance (C) of substance X was calculated as C_X_=U_X_ x UO/P_X_, where U_X_ denotes concentration of x in urine and P_X_ denotes concentration of x in plasma and UO is urine excretion rate. FE_Na_ was determined according to the following formula: FE_Na_ = 100 x ((u-Na x p-Cr))/((p-Na x u-Cr)). Free water clearance was determined according to the following formula: C_H2O_ = UO-C_osm._

#### 3.1.2. AQP2 and ENaC in Urine

Urine samples were kept frozen at −20°C until assayed. U-AQP2 was determined by RIA as described in details previously [13, 14]. AQP2 standard and rabbit anti-AQP2 antibodies were a gift from Professor Soren Nielsen and Professor Robert Fenton, the Water and Salt Research Center, Aarhus University, Denmark. Iodination of ENaC*γ* was performed by the chloramine *T* method. Samples and standard were incubated during 24 hours, and then the tracer was added. After further 4 hours, the reaction was stopped by gamma globulin and polyethylene glycol. After centrifugation, the precipitate (bound fraction) was counted in a gamma counter. The minimal detection level was 32 pg/tube. Coefficients of variation: 11.7% (interassay) and 5.9% (intra-assay). U-ENaC*γ* was measured by RIA as described in details previously [15, 16]. ENaC*γ* was synthesized and purchased by Lofstrand, Gaithersburg, Maryland, USA. It is a protein and part of the epithelial sodium channel in the distal part of the nephron. The ENaC*γ* antibody was a gift from Professor Soren Nielsen and Professor Robert Fenton, the Water and Salt Center, Aarhus University. Iodination of ENaC*γ* was performed by the chloramine *T* method. Samples and standard were incubated during 24 hours, and then the tracer was added. After further 4 hours of incubation, gammaglobulin and polyethylene glycol was added. The mixture was centrifuged, and the precipitate was counted in a gamma counter. The minimal detection level was 35 pg/tube. Coefficients of variation was 10% at a mean level of 338 pg/tube (inter-assay), 9 % at a mean level of 743 pg/tube (interassay), 5.0 % in the range 125–135 pg/tube (intra-assay), and 5.6 % in the range 290–380 pg/tube (intra-assay).

#### 3.1.3. Vasoactive Hormones in Plasma

Blood samples for measurements of vasoactive hormones were centrifuged for 10 minutes at 2200 G and 4°C. Plasma was separated from blood cells and kept frozen until assayed. P-AVP and p-AngII were extracted from plasma with C_18_ Sep-Pak (Waters Corporation, Milford, MA, USA) and determined by radioimmunoassay (RIA) as previously described [13, 14]. The antibodies against AVP were a gift from Professor Jacques Dürr (Miami, FL, USA). The minimal detection level was 0.5 pmol/L. The coefficients of variation were 13% (inter-assay) and 9% (intra-assay). Antibodies against AngII were obtained from the Department of Clinical Physiology, Glostrup Hospital, Denmark. The minimal detection level was 2 pmol/L. The coefficients of variation were 12% (interassay) and 8% (intra-assay). P-Aldo was determined by a RIA kit from Demeditec Diagnostics GmbH, Kiel, Germany. The minimal detection level was 14.8 pg/mL. Coefficients of variations were 10.2% (interassay) and 11.9% (intra-assay). PRC was determined by a RIA kit (CIS Bio International, Gif-Sur-Yvette Cedex, France). The minimal detection level was 1 pg/mL. The coefficients of variations were 4.1% (interassay) and 1.8% (intra-assay).

#### 3.1.4. Other Biochemical Measurements

U-Osm and p-Osm were measured using A_2_O Advanced Automated Osmometer (Advanced Instruments, MA, USA). Plasma concentration of sodium, potassium, albumin, hemoglobin, leukocytes, platelets, creatinine, bilirubin, ALAT, alkaline phosphatase, cholesterol, calcium, phosphate, PTH, TSH, and HbA1c was measured using routine methods at the Department of Clinical Biochemistry, Holstebro Hospital, Denmark.

#### 3.1.5. Brachial and Central Blood Pressure

BP was measured every twenty minutes throughout the examination day. Heart rate, bBP, cBP, mean arterial pressure (MAP), PWV, and AIx were measured using an oscillometric device (Mobil-O-Graph® PWA).

#### 3.1.6. Statistics

Statistical analyses were performed using IBM SPSS statistics version 20 (SPSS Inc., Chicago, IL, USA). For comparison between and within the three groups, we used a general linear Model with Greenhouse-Geisser correction for violating the assumption of sphericity with repeated-measures ANOVA. Data were tested for normal distribution. For comparison between two groups, we used a paired or unpaired *t* test, when data showed normal distribution. For data which did not show normal distribution, we used Mann-Whitney' s *U* test for unpaired data and Wilcoxon's signed rank test for paired data. Statistical significance was set at <0.05 in all analyses. Data with normal distribution are reported as mean ± SD, and data with nonnormal distribution are reported as medians with 25% and 75% percentiles in brackets.

## 4. Results

### 4.1. Demographics

Baseline demographics and clinical characteristics are presented in Table 1. Twenty ADPKD patients with chronic kidney disease stages I–IV were allocated to the study. One patient was excluded due to problems with venepuncture. One patient was excluded due to withdrawal of consent. Thus, 18 patients were included in Group 1. Twenty non-ADPKD patients with CKD stages I-IV were allocated to the study. One patient was excluded due to side effects of the background antihypertensive medicine. Three patients were excluded due to withdrawal of consent. Thus, 16 patients were included in Group 2. Twenty healthy controls were included in the study. Two participants were excluded due to withdrawal of consent. Thus, 18 participants were included in Group 3.

Al three groups had similar age (ADPKD patients had a median age of 53 years, non-ADPKD patients 56 years, healthy controls 57 years *p*=0.469 between ADPKD and non-ADPKD patients, *p*=0.393 between ADPKD patients and healthy controls, and *p*=0.863 between non-ADPKD patients and healthy controls). ADPKD patients and non-ADPKD patients had similar eGFR (69 ± 27 ml/min/17.3 m^2^ and 63 ± 33 ml/min/1.73 m^2^, *p*=0.566), but both patient groups had a lower eGFR compared to healthy controls (87 ± 13 ml/min/1.73 m^2^, *p*=0.013 and *p*=0.011, respectively). Among ADPKD patients, 12 received metoprolol, 14 amlodipine, and 2 no antihypertensive treatment. Among non-ADPKD patients, 5 received metoprolol, 5 amlodipine, and 6 no antihypertensive treatment. Overall, more antihypertensive treatment was needed in the ADPKD group.

In Group 2, 10 patients had a renal biopsy and 6 patients had no biopsy. The primary kidney disease was chronic nonspecified glomerulonephritis in 5 patients, chronic interstitial nephritis in 2 patients, and focal segmental glomerulosclerosis (FSGS) in 3 patients.

### 4.2. Weight Increase, Water Intake, and Total Urine Output

The three groups' water intake was similar (1699 ± 437 ml in ADPKD patients vs. 1497 ± 253 ml in non-ADPKD patients, *p*=0.106, and 1472 ± 189 ml in healthy controls, *p*=0.055 compared to ADPKD patients, and *p*=0.748 compared to non-ADPKD patients).

All three groups had a similar increase in body weight one hour after water intake compared to baseline (the increase was 1.4 ± 0.5 kg in ADPKD patients vs. 1.5 ± 0.3 kg in non-ADPKD patients, *p*=0.727 and 1.3 ± 0.3 kg in healthy controls, *p*=0.676 compared to ADPKD patients, and *p*=0.288 compared to non-ADPKD patients). Total urine output in period 3–7 was 1827 ± 553 ml in ADPKD patients, 1568 ± 370 ml in non-ADPKD patients, and 1739 ± 397 ml in healthy controls (*p*=0.122 when comparing ADPKD with non-ADPKD patients, *p*=0.585 when comparing ADPKD with healthy controls, and *p*=0.205 when comparing non-ADPKD with healthy controls).

### 4.3. Renal Water Excretion

**Figure 1:**
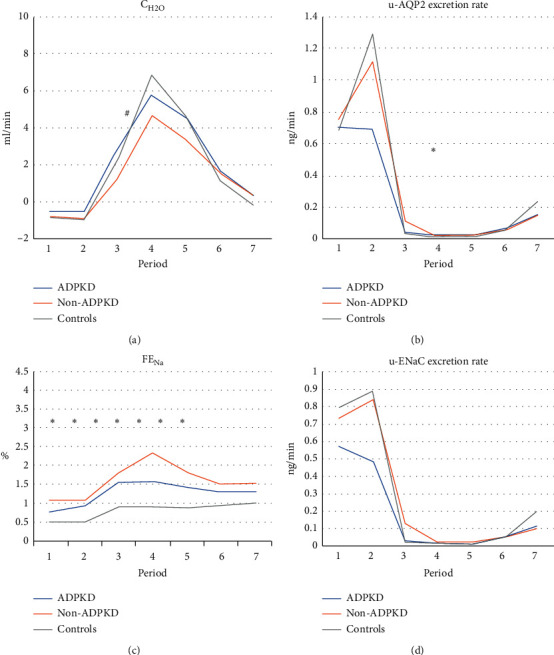
CH2O (a), u-AQP2 excretion rate (b), FENa (c), and u-ENaC excritoin rate (d) during urine dilution test in patients with adult dominant polycystic kidney disease (ADPKD), patients with non-ADPKD kidney disease (non-ADPKD), and healthy control subjects (controls). An oral water load of 20 ml/body weight was given immediately after Period 2. Urine was collected in seven periods. Period 1 (24 hours): from 07.30 AM the day before arrival to the laboratory to 07.30 AM on the examination day. Period 2 (one hour): 07.30–08.30 AM. Period 3 (one hour): 08.30–09.30 AM. Period 4 (one hour): 09.30–11.30 AM. Period 5 (one hour): 10.30–11.30 AM. Period 6 (one hour): 11.30–00.30 PM. Period 7 (on hour): 00.30–01.30 PM. Values represent mean. ^#^*p* < 0.005 between ADPKD and Non-ADPKD patients. (a). Values represent median ^*∗*^*p* < 0.005 between ADPKD and/or non-ADPKD patients and healthy controls (b). Values represent mean. ^*∗*^*p* < 0.005 between ADPKD and/or non-ADPKD patients and healthy controls (c). Values represent median ^*∗*^*p* < 0.005 between ADPKD and/or non-ADPKD patients and healthy controls (d).

C_H2O,_ UO, u-Osm, p-Osm, and u-AQP2 are presented in Table 2. Means of C_H2O_ and u-AQP2 are given in Figures 1(a) and 1(b).

For C_H2O_, comparison within groups showed that C_H2O_ increased rapidly after water load and peaked in Period 4 in all groups. Comparison between the three groups showed that in Period 3, the ADPKD patients had an increased C_H2O_ compared to non-ADPKD patients (2.97 ± 2.42 ml/min in ADPKD patients vs. 1.31 ± 1.50 ml/min in non-ADPKD patients, *p*=0.029), but no difference existed between ADPKD or non-ADPKD patients and controls (*p*=0.526 and *p*=0.177, respectively). In Period 4, we saw a lower increase in non-ADPKD patients compared to healthy controls (5.6 ± 3.7 ml/min in non-ADPKD patients vs. 8.3 ± 3.5 ml/min in healthy controls, *p*=0.043). There was no significant difference between non-ADPKD and ADPKD patients (*p*=0.605) or ADPKD patients and healthy controls (*p*=0.175). In Period 7, all three groups had similar C_H2O_ levels compared to baseline (C_H2O_ differed with 0.9 ± 1.9 within ADPKD patients, 1.2 ± 1.4 ml/min within non-ADPKD patients and 0.6 ± 1.4 ml/min within healthy controls, *p* > 0.05 for all).

For UO, comparison within groups showed that UO increased rapidly after water loading and peaked in Period 4 in all groups. Comparison between groups showed that ADPKD patients had an increased UO compared to non-ADPKD patients in Period 2 (1.89 ± 1.61 ml/min vs. 0.99 ± 0.36 ml/min, *p*=0.039). Otherwise, the three groups had a similar UO throughout the examination day (*p* > 0.05 for all).

For u-Osm, comparison within groups showed that u-Osm decreased after water load in all three groups, and the maximal decrease was in Period 4 from a level around 500 mosmol/kg to around 100 mosmol/kg. Comparison between groups showed that in Period 2, u-Osm was lower in ADPKD patients compared to non-ADPKD patients and healthy controls (463 ± 149 mosmol/kg in ADPKD patients vs. 595 ± 187 mosmol/kg in non-ADPKD patients, *p*=0.028, and vs. 639 ± 209 mosmol/kg in healthy controls, *p*=0.007). In Period 3, u-Osm remained lower in ADPKD than in non-ADPKD patients (*p*=0.023). In Period 7, u-Osm was significantly more decreased compared to baseline (Period 2) in ADPKD patients than in non-ADPKD patients (the decrease between Periods 2 and 7 was 150 ± 175 mosmol/kg in ADPKD patients vs. 288 ± 186 mosmol/kg in non-ADPKD patients, *p*=0.034, and 220 ± 248 mosmol/kg in healthy controls, *p*=0.341 compared to ADPKD patients and *p*=0.383 compared to non-ADPKD patients, respectively).

Comparison within groups showed that p-Osm and p-AVP were similar between the three groups throughout the examination day (*p*=0.758 and *p*=0.866, respectively). In addition, p-Osm and p-AVP did not deviate significantly during the test although there was a clear tendency to a decrease in p-Osm (*p*=0.075 for p-Osm and *p*=0.243 for p-AVP). However, the decrease in p-Osm between Period 2 and Period 6 was more pronounced in the two patient groups compared with healthy controls (4.4 ± 2.9 mosmol/kg in ADPKD patients and 4.4 ± 3.2 mosmol/kg in non-ADPKD patients vs. 2.2 ± 2.5 mosmol/kg in healthy controls, *p*=0.038 and *p*=0.025, respectively).

The U-AQP2 excretion rate decreased to a very low level in all three groups with the lowest level in Periods 4 and 5. There was a very small difference between the groups in Period 4, where non-ADPKD patients had an higher u-AQP2 excretion rate compared to healthy controls (0.02 [0.01; 0.09] vs. 0.01 [0.00; 0.02], *p*=0.023).

### 4.4. Renal Sodium Excretion

FE_Na_ and u-ENaC are given in Table 3, and the means are given in Figures 1(c) and 1(d).

Comparison within groups showed an increase in FE_Na_ to a maximum level in Period 4 in all groups. Although a considerable variation existed between the groups, the relative increase was approximately the same in all three groups, i.e., a doubling. U-ENaC decreased to very low levels in all groups in Period 4 and remained low during the following periods.

Comparison between groups showed that FE_Na_ was increased in the two patients groups compared to healthy controls in Period 2 (0.92 ± 0.60 % in ADPKD patients, 1.09 ± 0.72 in non-ADPKD patients vs. 0.51 ± 0.24 % in healthy controls, *p*=0.013 and *p*=0.006, respectively). This difference remained during the dilution test.

The U-ENaC excretion rate decreased to a very low level in all three groups with the lowest level in Periods 4 and 5. However, ADPKD patients had a lower decrease in u-ENaC excretion rate compared to non-ADPKD patients and healthy controls, when comparing the decrease between Period 2 and Period 4 (0.5 [0.3; 1.0] ng/min in ADPKD patients vs. 0.8 [0.6; 1.5] ng/min in non-ADPKD patients, *p*=0.042 and 0.9 [0.6; 2.9] ng/min in healthy controls, *p*=0.029).

### 4.5. Renal Albumin Excretion and Creatinine Clearance

C_Cr_ and u-Alb are presented in Table 3.

C_Cr_ was similar throughout the examination day in all three groups (*p*=0.204). C_Cr_ was decreased in non-ADPKD patients compared to healthy controls thorough the examination day (*p* < 0.05 in all periods), but not when comparing ADPKD patients to healthy controls (*p*=*NS* in all periods). However, we did not measure any significant difference in C_Cr_ between ADPKD patients and non-ADPKD patients thorough the examination day (*p*=*NS* in all periods).

After water loading, albumin excretion rate increased in the two patient groups compared to healthy controls (0.002 [0.000; 0.007] mg/min in ADPKD, 0.012 [0.002; 0.041] mg/min in non-ADPKD vs. 0.000 [0.000; 0.001] mg/min in healthy controls, *p*=0.05 and *p* < 0.001, respectively). Non-ADPKD patients had an increased albumin excretion rate compared to ADPKD patients at baseline (*p*=0.004) which prevailed after water loading.

### 4.6. Vasoactive Hormones

AVP, PRC, Aldo, and AngII are presented in Table 4.

There were no significant differences in p-AVP, PRC and p-AngII either within or between the three groups during the water dilution test.

P-Aldo was increased in ADPKD patients compared to healthy controls at baseline but not compared to non-ADPKD patients (250 ± 115 in ADPKD patients vs. 167 ± 49 in healthy controls, *p*=0.009 and 203 ± 147 in non-ADPKD patients, *p*=0.301). The increased p-Aldo in ADPKD patients compared to healthy controls remained after water load (*p*=0.018).

### 4.7. Systemic Haemodynamics

Systolic bBP, diastolic bBP, MAP, heart rate, systolic cBP, diastolic cBP, Aix, and PWV are presented in Table 5.

Systolic bBP was higher at baseline (Period 2) in the two patient groups compared to healthy controls (129 ± 11 mm Hg in ADPKD patients vs. 115 ± 13 mm Hg in healthy controls, *p*=0.002 and 125 ± 14 mm Hg in non-ADPKD patients, *p*=0.034). This difference remained throughout the examination day (*p* < 0.05 for all).

At baseline (Period 2), ADPKD but not non-ADPKD patients had an increased systolic cBP compared to healthy controls (120 ± 13 mm Hg in ADPKD patients vs. 107 ± 13 mm Hg in healthy controls, *p*=0.006 and 115 ± 13 mm Hg in non-ADPKD patients, *p*=0.097). One hour after water load (Period 3), ADPKD patients had an increased systolic cBP compared to both non-ADPKD (*p*=0.039) and healthy controls (*p* < 0.001). However, during the rest of the test (Periods 4–7), there was no significant difference between the two patient groups (*p*=0.159 in Period 4, *p*=0.265 in Period 5, *p*=0.464 in Period 6, and *p*=0.901 in Period7).

MAP was significantly higher in the patient groups than in controls, except for Period 2 in which a significant difference between non-ADPKD patients and healthy controls was not measured (*p*=0.053), most likely by chance.

Diastolic bBP and diastolic cBP showed a similar pattern with an increased diastolic BP in ADPKD patients compared to healthy control throughout the examination day (*p* < 0.05 for all) and increased diastolic BP in ADPKD patients compared to non-ADPKD after water load (Periods 4 and 5, *p* < 0.05).

AIx and PWV did not change during the examination day (*p*=0.212 and *p*=0.852, respectively), and there were similar PWV in the three groups (*p*=0.100).

## 5. Discussion

The present paper compares urine dilution ability, tubular function, vasoactive hormones, and systemic hemodynamics in ADPKD patients, non-ADPKD patients with chronic kidney disease, and healthy controls after an oral water load. The major findings were that all three groups had qualitatively the same response to water loading, but quantitatively, healthy controls had the highest urine diluting capacity and non-ADPKD patients the lowest with ADPKD patients in between.

The final regulation of renal water and sodium excretion occurs in the distal part of the nephron. The main tubular transporters of water and sodium are the aquaporin2 water channels regulated by vasopressin, and the epithelial sodium channels regulated by mineralocorticoids via the mineralocorticoid receptor, respectively. Abnormal function is well documented of both the osmoregulatory system and the renin-angiotensin-aldosterone system in heart failure, liver disease, and chronic renal disease. In the present study, our focus has been to measure the distal tubular function during water loading in ADPKD and non-ADPKD with mild to moderate reduced renal function. We used modern methods to reflect the activity in the aquaporin2 water channels (u-AQP2) and the epithelial sodium channels (u-ENaC*γ*). We wanted to clarify whether the different pathophysiological mechanisms in progression of kidney disease in ADPKD and non-ADPKD also resulted in different function of the distal tubules regarding sodium and water transportation.

### 5.1. Renal Water Excretion

After water loading, the ADPKD patients had an increased C_H2O_ compared to non-ADPKD patients. This means a faster elimination of excess water in ADPKD patients than in non-ADPKD patients. Although urine diluting ability tended to be higher in healthy controls, it did not deviate significantly from patients with ADPKD, but it was significantly better than in non-ADPKD patients. Not surprisingly, these results are in good agreement with measurements of u-Osm, which was reduced to approximately the same degree in ADPKD patients and healthy controls. A decreased urine diluting ability in patients with reduced renal function is in accordance with that in the earlier studies [4, 5].

A previous study showed a decrease in p-Osm in CKD-patients after oral water loading with 20 ml/body weight [5]. In the present study, we also measured a larger decrease in p-Osm in the two patient groups compared to the controls. This confirms the reduced and slower ability to eliminate water in kidney disease.

U-AQP2 excretion rate decreased pronouncedly in all three groups after water loading to a very low level in all. Since u-AQP2 reflects water transport in the distal part of the nephron, our measurement is in good agreement with a very low or transitorily ceased water absorption in the final diluting part of the tubular system. We measured a small but significantly increased u-AQP2 in the non-ADPKD patients compared to healthy controls. This lack of reduction in u-AQP2 is in agreement with a reduced ability to increase water excretion in this group of patients as also demonstrated in measurements of free water clearance. The u-AQP2 levels were very low in all three groups during the test, and in cannot be excluded that the difference is by chance. Although eGFR was the same in the two groups of patients, there was a tendency to a lower creatinine clearance in the non-ADPKD patients compared to ADPKD patients. Thus, the lower u-AQP2 in non-ADPKD patients may reflect a slightly lower renal function in non-ADPKD patients and consequently a lower ability to reduce u-AQP2.

### 5.2. Renal Sodium Excretion

FE_Na_ increased in all three groups during the urine diluting test, and as expected, it was at a significantly higher level in the two patient groups than in the controls. U-ENaC reflects sodium transport via the epithelial sodium channels in the distal part of the nephron. Although u-ENaC was reduced in all three groups, the reduction was more pronounced in healthy controls and ADPKD patients than in non-ADPKD patients. As for u-AQP2, this suggests a lower tubular response in non-ADPKD patients compared with the other groups. Of course, this difference may be by chance due to the very low levels of u-ENaC, but it could also be attributed to the fact that non-ADPKD had the lowest renal function, when estimated by creatinine clearance as for the low level of u-AQP2.

### 5.3. Creatinine Clearance and Albumin Excretion

The study was designed to have two patient groups with the same degree of reduction in renal function. This was also obtained when looked at eGFR. However, during measurement of creatinine clearance during the study, the renal function was significantly lower in non-ADPKD than in control subjects and tended to be lower than in ADPKD patients. A lower creatinine clearance in the non-ADPKD group may contribute to the measured lower urine diluting ability in the non-ADPKD group compared to the other groups. This may also be in good agreement with the slightly higher urine excretion of albumin in non-ADPKD patients, but the levels of urine albumin excretion were very low in all three groups from Period 3 and during the test. Thus, conclusions cannot be drawn about albumin excretion during the diluting test.

### 5.4. Vasoactive Hormones

We found no significant changes in PRC, p-AngII, or p-Aldo which could explain the changes in urinary excretion of water and sodium between the groups during the urine dilution test. P-Aldo was increased in ADPKD patients compared to healthy controls at baseline and after water loading. This is in agreement with previous studies that have showed activation of the RAAS-system in CKD, including ADPKD [17–19]. P-AVP was expected to fall during water loading. We measured a tendency to a reduction p-AVP in both healthy controls and ADPKD patients, but no significant changes, which is in agreement with a previous study with healthy subjects [20]. The reason might be that the subjects did not thirst up to the water loading test. Thus, vasopressin secretion was not stimulated before the test. Another explanation might be that the patients had on average a relatively well preserved renal function with eGFR in the range 60–70 ml/min/1.73 m^2^.

### 5.5. Systemic Haemodynamics

Blood pressure is a known factor to influence kidney function [21]. Blood pressure was slightly elevated in the two groups of patients. However, we measured no clinical significant changes either within or between the three groups regarding brachial or central blood pressure. Thus, a possible influence of blood pressure on the results of the urine dilution test is estimated to be absent or minimal.

## 6. Strengths and Limitations

The three groups had similar age and gender segregation and digested similar amount of water during the water loading. Furthermore, the two patient groups had similar eGFR, which was significantly decreased compared with healthy controls. This match is a strength in our study as well as the length and uniform procedure of the dilution test. However, during the study, the values of creatinine clearances tended to be systematically lower in non-ADPKD than in ADPKD patients. Although no significant differences existed between the two groups of patients, we must accept this lack of concordance between eGFR and creatinine clearance as a weakness. We did not find it ethically justified to discontinue antihypertensive medication. Therefore, patients received antihypertensive treatment during the study, but it was standardized to only metoprolol and/or amlodipine, and a possible influence of antihypertensive treatment on the results is estimated to be absent or minimal during the urine dilution test.

## 7. Conclusion

ADPKD patients had a higher free water clearance than non-ADPKD patients during a water loading test, and urinary excretion of both AQP2 and ENaC was more rapidly reduced than in non-ADPKD patients. Thus, urine-diluting capacity may be better preserved in patients with ADPKD than in patients with other chronic kidney diseases having the same degree of reduction in renal function.

## Figures and Tables

**Table 1:** Baseline demographics of the participants in the study.

	ADPKD patients	Non-ADPKD	Controls
Number of subjects (*n*)	18	16	18
Age (years)	55 ± 13	56 ± 17	55 ± 15
Gender (men/women)	9/9	9/7	8/10
Office systolic bBP (mmHg)	137 ± 16^*∗*^	130 ± 16	125 ± 10
Office diastolic bBP (mmHg)	79 ± 10^#^	72 ± 9	74 ± 7
eGFR (ml/min/17.3 m^2^)	69 ± 27^*∗*^	63 ± 33^*∗*^	87 ± 13
p-Creatinine (*µ*mol/l)	107 ± 44^*∗*^	124 ± 57^*∗*^	76 ± 11
u-Albumin (mg/l)	17 [8; 50]^*∗*#^	81 [26; 283]^*∗*^	8 [3, 11]

Patients with adult dominant polycystic kidney disease (ADPKD), patients with non-ADPKD kidney disease (non-ADPKD), and healthy control subjects (controls). Values represent *n* in either group or mean ± SD or median with 25% and 75% percentiles in brackets. ^*∗*^*p* < 0.05 between ADPKD or non-ADPKD patients and healthy controls. ^#^*p* < 0.05 between ADPKD and non-ADPKD patients. ADPKD = patients with adult dominant polycystic kidney disease; non-ADPKD = patients with non-ADPKD kidney disease; controls = healthy control subjects. bBP = brachial blood pressure; eGFR = estimated glomerular filtration rate.

**Table 2:** Renal water excretion during the water loading test in patients with adult dominant polycystic kidney disease (ADPKD), patients with non-ADPKD kidney disease (non-ADPKD), and healthy control subjects (controls).

Time		Baseline Period 1 07.30 AM-07.30 AM	Baseline Period 2 07.30 AM-08.30 AM	Period 3 08.30 AM-09.30 AM	Period 4 09.30 AM-10.30 AM	Period 5 10.30 AM-11.30 AM	Period 6 11.30 AM-00.30 PM	Period 7 00.30 PM-01.30 PM
C_H20_ (ml/min)	ADPKD Non-ADPKD Controls	(−0.52 ± 0.91) (−0.79 ± 0.81) (−0.83 ± 0.45)	(−0.52 ± 0.92) (−0.85 ± 0.61 (−0.97 ± 0.70	2.97 ± 2.42^#^ 1.31 ± 1.50 2.40 ± 2.71	5.80 ± 4.09 4.71 ± 3.38 6.89 ± 3.50	4.55 ± 2.25 3.41 ± 1.86 4.68 ± 2.27	1.77 ± 2.27 1.59 ± 1.47 1.22 ± 1.57	0.40 ± 1.71 0.37 ± 1.34 (-0.14 ± 1.59

Comparison within groups		0.200						

Comparison between groups		0.023						

U-Osm (mosmol/kg)	ADPKD Non-ADPKD Controls	485.6 ± 136.0 568.8 ± 251.6 538.3 ± 162.4	463.4 ± 149.1^*∗*#^ 595.9 ± 187.3 639.5 ± 209.4	199.7 ± 87.9^#^ 287.3 ± 125.0 235.4 ± 145.2	96.1 ± 25.7 118.5 ± 43.8 95.9 ± 41.7	111.0 ± 38.1 126.1 ± 28.4 113.7 ± 60.8	209.7 ± 106.6 207.7 ± 92.9 231.8 ± 143.5	313.2 ± 136.6 307.8 ± 138.3 403.7 ± 182.7

Comparison within groups		0.065						

Comparison between groups		0.055						

P-Osm (mosmol/kg)	ADPKD Non-ADPKD Controls	X	292 ± 4 293 ± 6 292 ± 3	286 ± 5 287 ± 5 285 ± 4	286 ± 3 287 ± 4 286 ± 3	288 ± 4 288 ± 5 289 ± 4	288 ± 4 289 ± 5 290 ± 4	288 ± 4 289 ± 5 290 ± 4

Comparison within groups		X	0.075					

Comparison between groups		X	0.758					

U-AQP2 excretion rate (ng/min)	ADPKD Non-ADPKD Controls	0.70 [0.38; 1.20] 0.75 [0.49; 1.58] 0.69 [0.45; 1.01]	0.69 [0.39; 1.32] 1.12 [0.66; 1.45] 1.29 [0.50; 2.34]	0.04 [0.02; 0.10] 0.11 [0.03; 0.17] 0.03 [0.02; 0.13]	0.02 [0.01; 0.06] 0.02 [0.01; 0.09]^*∗*^ 0.01 [0.00; 0.02]	0.02 [0.01; 0.03] 0.02 [0.01; 0.05] 0.01 [0.01; 0.03]	0.06 [0.03; 0.13] 0.05 [0.03; 0.10] 0.05 [0.03; 0.08]	0.15 [0.06; 0.27] 0.15 [0.05; 0.20] 0.23 [0.13; 0.48]

Kruskal-Wallis test		0.872	0.316	0.213	0.087	0.287	0.895	0.159

UO (ml/min)	ADPKD Non-ADPKD Controls	1.57 ± 1.07 1.13 ± 0.39 1.16 ± 0.45	1.89 ± 1.61^#^ 0.99 ± 0.36 1.08 ± 0.63	6.50 ± 3.77 4.47 ± 2.18 5.55 ± 3.31	9.09 ± 4.89 8.06 ± 3.63 9.92 ± 4.13	7.33 ± 2.52 6.09 ± 2.35 7.35 ± 2.27	4.29 ± 2.55 4.07 ± 1.42 3.71 ± 1.43	2.80 ± 1.87 2.89 ± 1.26 2.35 ± 1.66

Comparison within groups		0.299						

Comparison between groups		0.157						

An oral water load of 20 ml/body weight was given immediately after period 2. Urine was collected in seven periods. Period 1 (24 hours): from 07.30 AM the day before arrival to the laboratory to 07.30 AM on the examination day. Period 2 (one hour): 07.30–08.30 AM. Period 3 (one hour): 08.30–09.30 AM. Period 4 (one hour): 09.30–11.30 AM. Period 5 (one hour): 10.30–11.30 AM. Period 6 (one hour): 11.30–00.30 PM. Period 7 (on hour): 00.30–01.30 PM. A general linear model with Greenhouse-Geisser correction was used for comparison within and between the three groups. Values represent mean ± SD or median with 25% and 75% percentiles in brackets ∗*p* < 0.05 between ADPKD or Non-ADPKD patients and controls. #*p* < 0.05 between ADPKD and Non-ADPKD patients. CH2O = free water clearance. U-AQP2 = urinary aquaporin-2, Osm = osmolality. UO = urine output.

**Table 3:** Kidney tubular function during urine dilution test in patients with adult dominant polycystic kidney disease (ADPKD), patients with non-ADPKD kidney disease (non-ADPKD), and healthy control subjects (controls).

		Baseline Period 1 07.30 AM-07.30 AM	Baseline Period 2 07.30 AM-08.30 AM	Period 3 08.30 AM-09.30 AM	Period 4 09.30 AM-10.30AM	Period 5 10.30 AM-11.30 AM	Period 6 11.30 AM-00.30 PM	Period 7 00.30 PM-01.30 PM
C_Cr_ (ml/min)	ADPKD Non-ADPKD Controls	107.9 ± 49.5 85.3 ± 49.0^*∗*^ 128.1 ± 33.1	111.8 ± 52.3 83.3 ± 45.1^*∗*^ 137.1 ± 35.8	121.5 ± 44.5 97.0 ± 48.4^*∗*^ 141.7 ± 30.2	120.0 ± 44.2 99.1 ± 47.1^*∗*^ 135.9 ± 27.0	107.8 ± 42.2 89.4 ± 44.5^*∗*^ 127.3 ± 23.6	105.3 ± 43.3 93.5 ± 47.9^*∗*^ 123.7 ± 32.7	106.0 ± 48.4 96.8 ± 50.8^*∗*^ 133.0 ± 33.5

Comparison within groups		0.204						

Comparison between groups		0.018						

FE_Na_ (%)	ADPKD Non-ADPKD Controls	0.79 ± 0.33^*∗*^ 1.08 ± 0.59^*∗*^ 0.50 ± 0.17	0.92 ± 0.60^*∗*^ 1.09 ± 0.72^*∗*^ 0.51 ± 0.24	1.56 ± 0.93^*∗*^ 1.83 ± 1.14^*∗*^ 0.90 ± 0.36	1.58 ± 0.82^*∗*^ 2.34 ± 1.71^*∗*^ 0.90 ± 0.41	1.42 ± 0.77^*∗*^ 1.84 ± 1.30^*∗*^ 0.88 ± 0.36	1.31 ± 0.62^*∗*^ 1.50 ± 1.03 0.96 ± 0.29	1.31 ± 0.58 1.55 ± 0.98^*∗*^ 1.00 ± 0.35

Comparison within groups		0.099						

Comparison between groups		0.002						

Alb excretion rate (mg/min)	ADPKD Non-ADPKD Controls	0.017 [0.001; 0.063]^*∗*#^ 0.088 [0.032; 0.280]^*∗*^ 0.003 [0.002; 0.007]	0.012 [0.005; 0.057]^#^ 0.066 [0.036; 0.470]^*∗*^ 0.007 [0.002; 0.018]	0.002 [0.000; 0.007]^*∗*#^ 0.012 [0.002; 0.041]^*∗*^ 0.000 [0.000; 0.001]	0.001 [0.000; 0.003]^*∗*^ 0.002 [0.001; 0.010]^*∗*^ 0.000 [0.000; 0.000]	0.000 [0.000; 0.002]^*∗*#^ 0.003 [0.000; 0.015]^*∗*^ 0.000 [0.000; 0.000]	0.001 [0.000; 0.004]^*∗*#^ 0.005 [0.001; 0.025]^*∗*^ 0.000 [0.000; 0.001]	0.002 [0.001; 0.006]^*∗*#^ 0.013 [0.002; 0.046]^*∗*^ 0.001 [0.000; 0.002]

Kruskal-Wallis test		<0.001	<0.001	<0.001	<0.001	<0.001	<0.001	0.002

U-ENaC excretion rate (ng/min)	ADPKD Non-ADPKD Controls	0.57 [0.42; 0.99] 0.74 [0.58; 1.35 0.80 [0.56; 1.15]	0.49 [0.43; 1.1] 0.84 [0.67; 1.56] 0.89 [0.60; 2.9]	0.03 [0.02; 0.08] 0.13 [0.03; 0.20] 0.02 [0.01; 0.09]	0.01 [0.00; 0.05] 0.02 [0.01; 0.08] 0.01 [0.00; 0.01]	0.01 [0.01; 0.02] 0.02 [0.01; 0.04] 0.01 [0.01; 0.03]	0.05 [0.02; 0.10] 0.05 [0.03; 011] 0.05 [0.03; 0.08]	0.11 [0.07; 0.20] 0.10 [0.05; 0.19] 0.20 [0.07; 0.36]

Kruskal-Wallis test		0.590	0.074	0.143	0.195	0.086	0.954	0.292

An oral water load of 20 ml/body weight was given immediately after period 2. Urine was collected in seven periods. Period 1 (24 hours): from 07.30 AM the day before arrival to the laboratory to 07.30 AM on the examination day. Period 2 (one hour): 07.30–08.30 AM. Period 3 one hour): 08.30–09.30 AM. Period 4 (one hour): 09.30–11.30 AM. Period 5 (one hour): 10.30–11.30 AM. Period 6 (one hour): 11.30–00.30 PM. Period 7 (on hour): 00.30–01.30 PM. A general linear model with Greenhouse-Geisser correction was used for comparison within and between the three groups. Values represent mean ± SD or median with 25% and 75% percentiles in brackets ∗*p* < 0.05 between ADPKD or Non-ADPKD patients and controls. #*p* < 0.05 between ADPKD and Non-ADPKD patients. *C*_Cr_ = creatinine clearance. Alb = albumin. FE_Na_ = fractional excretion of sodium. U-ENaC = urinary epithelial sodium channel.

**Table 4:** Hormones during urine dilution test in patients with adult dominant polycystic kidney disease (ADPKD), patients with non-ADPKD kidney disease (non-ADPKD), and healthy control subjects controls).

		Baseline Period 2	Period 4	Period 6	Period 7
AVP (pg/ml)	ADPKD Non-ADPKD Controls	0.3 ± 0.2 0.4 ± 0.3 0.3 ± 0.1	0.2 ± 0.1 0.3 ± 0.2 0.2 ± 0.1	0.3 ± 0.2 0.3 ± 0.3 0.3 ± 0.1	0.3 ± 0.2 0.3 ± 0.2 0.4 ± 0.2

Comparison within groups		0.243			

Comparison between groups		0.866			

PRC (pg/ml)	ADPKD Non-ADPKD Controls	7 [4, 9] 8 [5, 17]^*∗*^ 6 [4, 8]	6 [5, 8] 7 [5, 18] 6 [4, 7]	7 [5, 8] 7 [5, 19]^*∗*^ 5 [3, 7]	5 [4, 9] 6 [4, 19] 5 [3, 7]

Kruskal-Wallis test		0.136	0.297	0.059	0.550

AngII (pmol/l)	ADPKD Non-ADPKD Controls	6 ± 4 5 ± 3 5 ± 3	5 ± 3 5 ± 3 5 ± 4	5 ± 3 5 ± 2 6 ± 4	6 ± 4 6 ± 3 7 ± 4

Comparison within groups		0.024			

Comparison between groups		0.936			

Aldo (pg/ml)	ADPKD Non-ADPKD Controls	250 ± 115^*∗*^ 203 ± 147 167 ± 49	235 ± 104^*∗*^ 201 ± 140 154 ± 55	218 ± 134^*∗*^ 174 ± 106 120 ± 47	238 ± 139^*∗*^ 170 ± 92 136 ± 50

Comparison within groups		0.668			

Comparison between groups		0.025			

An oral water load of 20 ml/body weight was given immediately after period 2. Urine was collected in seven periods. Period 1 (24 hours): from 07.30 AM the day before arrival to the laboratory to 07.30 AM on the examination day. Period 2 (one hour): 07.30–08.30 AM. Period 3 (one hour): 08.30–09.30 AM. Period 4 (one hour): 09.30–11.30 AM. Period 5 (one hour): 10.30–11.30 AM. Period 6 (one hour): 11.30–00.30 PM. Period 7 (on hour): 00.30–01.30 PM. A general linear model with Greenhouse-Geisser correction was used for comparison within and between the three groups. Values represent mean ± SD or median with 25% and 75% percentiles in brackets ∗*p* < 0.05 between ADPKD or Non-ADPKD patients and controls. #*p* < 0.05 between ADPKD and Non-ADPKD patients. P-AVP = plasma vasopressin, PRC = plasma renin concentration. AngII = plasma angiotensin II concentration. Aldo = plasma aldosterone concentration.

**Table 5:** Hemodynamics during the urine dilution test in patients with adult dominant polycystic kidney disease (ADPKD), patients with non-ADPKD kidney disease (non-ADPKD), and healthy control subjects (controls).

		Baseline Period 2	Period 3	Period 4	Period 5	Period 6	Period 7
Systolic bBP (mm Hg)	ADPKD Non-ADPKD Controls	128.5 ± 11.3^*∗*^ 124.9 ± 14.0^*∗*^ 114.7 ± 12.7	141.0 ± 13.4^*∗*^ 138.5 ± 15.5^*∗*^ 126.1 ± 14.9	133.0 ± 11.8^*∗*^ 131.4 ± 14.1^*∗*^ 121.4 ± 13.2	128.9 ± 10.1^*∗*^ 129.2 ± 11.4^*∗*^ 121.7 ± 14.3	128.5 ± 11.0^*∗*^ 129.2 ± 11.7^*∗*^ 119.5 ± 13.6	132.1 ± 13.9^*∗*^ 133.0 ± 12.4^*∗*^ 121.7 ± 15.5

Comparison within groups		0,562					

Comparison between groups		<0.001					

Diastolic bBP (mm Hg)	ADPKD Non-ADPKD Controls	83.0 ± 8.3^*∗*^ 78.7 ± 8.3 74.4 ± 9.6	90.2 ± 10.3^*∗*^ 87.1 ± 7.5^*∗*^ 81.7 ± 11.7	86.1 ± 10.6^*∗*#^ 82.2 ± 7.4^*∗*^ 77.8 ± 10.7	83.4 ± 8.3^*∗*#^ 79.5 ± 6.3 76.7 ± 11.0	82.1 ± 7.8^*∗*^ 79.5 ± 8.4 75.8 ± 10.5	83.6 ± 8.7^*∗*^ 80.7 ± 7.1^*∗*^ 76.3 ± 11.7

Comparison within groups		0.306					

Comparison between groups		<0.001					

MAP (mm Hg)	ADPKD Non-ADPKD Controls	103.9 ± 8.8^*∗*^ 99.9 ± 10.3 92.8 ± 10.7	113.4 ± 10.5^*∗*^ 110.6 ± 10.3^*∗*^ 102.8 ± 12.8	107.5 ± 10.0^*∗*^ 104.7 ± 9.7^*∗*^ 96.9 ± 11.1	104.3 ± 8.2^*∗*^ 102.2 ± 7.8^*∗*^ 97.4 ± 12.1	103.3 ± 8.6^*∗*^ 102.2 ± 9.4^*∗*^ 95.8 ± 11.4	105.8 ± 9.7^*∗*^ 104.7 ± 8.9^*∗*^ 97.2 ± 12.9

Comparison within groups		0.347					

Comparison between groups		<0.001					

HR (beats/min)	ADPKD Non-ADPKD Controls	60.3 ± 9.5 58.5 ± 8.2 56.8 ± 7.4	60.4 ± 9.5^*∗*#^ 56.0 ± 6.5 54.8 ± 7.3	61.2 ± 10.1^*∗*#^ 57.7 ± 6.4 55.9 ± 7.4	62.1 ± 9.9^*∗*^ 59.0 ± 7.5 56.6 ± 6.9	61.3 ± 10.2 60.4 ± 7.3 58.8 ± 6.7	62.2 ± 9.0 62.2 ± 7.5 59.5 ± 6.9

Comparison within groups		0.671					

Comparison between groups		0.417					

Systolic cBP (mm Hg)	ADPKD Non-ADPKD Controls	119.6 ± 13.4^*∗*^ 114.6 ± 13.2 106.9 ± 12.6	131.3 ± 15.1^*∗*#^ 124.0 ± 12.5 117.8 ± 13.3	122.7 ± 13.0^*∗*^ 119.2 ± 11.2^*∗*^ 113.4 ± 13.0	120.0 ± 9.9^*∗*^ 17.8 ± 9.7 114.4 ± 14.5	119.2 ± 12.8^*∗*^ 117.3 ± 11.5 112.6 ± 13.8	122.7 ± 14.2^*∗*^ 122.3 ± 10.5^*∗*^ 115.2 ± 15.7

Comparison within groups		0.730					

Comparison between groups		<0.001					

Diastolic cBP (mm Hg)	ADPKD Non-ADPKD Controls	83.7 ± 8.1^*∗*^ 79.2 ± 8.9 75.2 ± 9.7	91.7 ± 9.8^*∗*^ 87.8 ± 7.4^*∗*^ 82.3 ± 12.1	87.1 ± 10.1^*∗*#^ 82.6 ± 7.5^*∗*^ 78.7 ± 10.9	84.4 ± 8.8^*∗*#^ 80.2 ± 6.0 77.2 ± 11.2	83.3 ± 7.9^*∗*^ 80.2 ± 8.6 76.5 ± 10.5	84.5 ± 8.6^*∗*^ 82.1 ± 7.5^*∗*^ 77.3 ± 12.0

Comparison within groups		0.770					

Comparison between groups		<0.001					

AIx (%)	ADPKD Non-ADPKD Controls	31.9 ± 9.8 32.7 ± 17.8 30.1 ± 13.0	33.7 ± 10.9 33.2 ± 13.7 31.0 ± 14.2	30.5 ± 13.5 31.1 ± 14.6 31.0 ± 13.5	30.1 ± 15.6 30.9 ± 15.5 29.7 ± 14.0	30.9 ± 14.1 30.3 ± 15.6 27.8 ± 13.3	30.4 ± 15.3 28.6 ± 14.9 25.9 ± 14.4

Comparison within groups		0.212					

Comparison between groups		0.013					

PWV (m/s)	ADPKD Non-ADPKD Controls	8.0 ± 1.7 8.2 ± 2.2 7.8 ± 1.8	8.4 ± 1.9 8.5 ± 2.2 8.1 ± 1.7	8.2 ± 1.8 8.2 ± 2.1 8.0 ± 1.7	8.1 ± 1.8 8.3 ± 2.0 8.0 ± 1.7	8.1 ± 1.8 8.2 ± 2.0 7.9 ± 1.7	8.2 ± 1.8 8.4 ± 2.1 8.0 ± 1.9

Comparison within groups		0.852					

Comparison between groups		0.100					

An oral water load of 20 ml/body weight was given immediately after period 2. Urine was collected in seven periods. Period 1 (24 hours): from 07.30 AM the day before arrival to the laboratory to 07.30 AM on the examination day. Period 2 (one hour): 07.30–08.30 AM. Period 3 (one hour): 08.30–09.30 AM. Period 4 (one hour): 09.30–11.30 AM. Period 5 (one hour): 10.30–11.30 AM. Period 6 (one hour): 11.30–00.30 PM. Period 7 (on hour): 00.30–01.30 PM. A general linear model with Greenhouse-Geisser correction was used for comparison within and between the three groups. Values represent mean ± SD or median with 25% and 75% percentiles in brackets ∗*p* < 0.05 between ADPKD or non-ADPKD patients and healthy controls. #*p* < 0.05 between ADPKD and non-ADPKD patients. bBP = brachial blood pressure, MAP = mean arterial pressure, HR = heart rate, cBP = central blood pressure, AIx = augmentation index, PWV = pulse wave velocity.

## Data Availability

The protocol and datasets used and/or analysed during the current study are available from the corresponding author on reasonable request.

## Abbreviations

ALAT: Alanine-amino-transaminase
AIx: Augmentation index
ADPKD: Autosomal dominant polycystic kidney disease
AQP2: Aquaporin-2
bBP: Brachial blood pressure
cBP: Central blood pressure
C_Cr_: Creatinine clearance
C_H2O_: Free water clearance
CKD: Chronic kidney disease
eGFR: Estimated glomerular filtration rate
ENaC: Epithelial sodium channel
FE_Na:_: Fractional excretion of sodium
HbA1c: Glycosylated hemoglobin A1c
MAP: Mean arterial pressure
Non-ADPKD: Patients with chronic kidney disease except ADPKD
*P*: *p* value
p-Aldo: Plasma aldosterone
p-AngII: Plasma angiotensin
p-AVP: Plasma vasopressin
P-Osm: Plasma osmolality
PRC: Plasma renin concentration
PTH: Parathyroid hormone
PWV: Pulse wave velocity
TSH: Thyroid-stimulating hormone
U-AQP2: Urine excretion of aquaporin 2 water channels
U-ENaC: Urine excretion of a fraction of the epithelial sodium channels
UO: Urine output
U-Osm: Urinary osmolality.

## References

[1] Jamison R. L., Oliver R. E. (1982). Disorders of urinary concentration and dilution. *The American Journal of Medicine*.

[2] Hoffert J. D., Fenton R. A., Moeller H. B. (2008). Vasopressin-stimulated increase in phosphorylation at Ser269Potentiates plasma membrane retention of aquaporin-2. *Journal of Biological Chemistry*.

[3] Nielsen S., Frøkiær J., Marples D., Kwon T.-H., Agre P., Knepper M. A. (2002). Aquaporins in the kidney: from molecules to medicine. *Physiological Reviews*.

[4] Davis B. B., Zenser T. V. (1993). Evaluation of renal concentrating and diluting ability. *Clinics in Laboratory Medicine*.

[5] Pedersen E. B., Thomsen I. M., Lauridsen T. G. (2010). Abnormal function of the vasopressin-cyclic-AMP-aquaporin2 axis during urine concentrating and diluting in patients with reduced renal function. A case control study. *BMC Nephrology*.

[6] Torres V. E., Harris P. C., Pirson Y. (2007). Autosomal dominant polycystic kidney disease. *The Lancet*.

[7] Schrier R. W., Brosnahan G., Cadnapaphornchai M. A. (2014). Predictors of autosomal dominant polycystic kidney disease progression. *Journal of the American Society of Nephrology*.

[8] Nakajima A., Lu Y., Kawano H., Horie S., Muto S. (2015). Association of arginine vasopressin surrogate marker urinary copeptin with severity of autosomal dominant polycystic kidney disease (ADPKD). *Clinical and Experimental Nephrology*.

[9] Wang X., Wu Y., Ward C. J., Harris P. C., Torres V. E. (2008). Vasopressin directly regulates cyst growth in polycystic kidney disease. *Journal of the American Society of Nephrology*.

[10] Yamaguchi T., Pelling J. C., Ramaswamy N. T. (2000). cAMP stimulates the in vitro proliferation of renal cyst epithelial cells by activating the extracellular signal-regulated kinase pathway11See Editorial by Grande, p. 1770. *Kidney International*.

[11] Malmberg M. H., Mose F. H., Pedersen E. B., Bech J. N. (2020). Urine concentration ability is reduced to the same degree in adult dominant polycystic kidney disease compared with other chronic kidney diseases in the same CKD-stage and lower THAN in healthy control subjects-a CASE control study. *BMC Nephrology*.

[12] Ravine D., Sheffield L., Danks D. M., Gibson R. N., Walker R. G., Kincaid-Smith P. (1994). Evaluation of ultrasonographic diagnostic criteria for autosomal dominant polycystic kidney disease 1. *The Lancet*.

[13] Pedersen R. S., Bentzen H., Bech J. N., Pedersen E. B. (2001). Effect of water deprivation and hypertonic saline infusion on urinary AQP2 excretion in healthy humans. *American Journal of Physiology-Renal Physiology*.

[14] Graffe C. C., Bech J. N., Pedersen E. B. (2012). Effect of high and low sodium intake on urinary aquaporin-2 excretion in healthy humans. *American Journal of Physiology-Renal Physiology*.

[15] Matthesen S. K., Larsen T., Lauridsen T. G. (2012). Effect of amiloride and spironolactone on renal tubular function, ambulatory blood pressure, and pulse wave velocity in healthy participants in a double-blinded, randomized, placebo-controlled, crossover trial. *Clinical and Experimental Hypertension*.

[16] Al Therwani S., Malmberg M. E. S., Rosenbaek J. B. (2017). Effect of tolvaptan on renal handling of water and sodium, GFR and central hemodynamics in autosomal dominant polycystic kidney disease during inhibition of the nitric oxide system: a randomized, placebo-controlled, double blind, crossover study. *BMC Nephrology*.

[17] Berl T., Katz F. H., Henrich W. L., de Torrente A., Schrier R. W. (1978). Role of aldosterone in the control of sodium excretion in patients with advanced chronic renal failure. *Kidney International*.

[18] Chapman A. B., Johnson A., Gabow P. A., Schrier R. W. (1990). The renin-angiotensin-aldosterone system and autosomal dominant polycystic kidney disease. *New England Journal of Medicine*.

[19] Hené R. J., Boer P., Koomans H. A., Dorhout Mees E. J. (1982). Plasma aldosterone concentrations in chronic renal disease. *Kidney International*.

[20] Pedersen R. S., Bentzen H., Bech J. N., Nyvad O., Pedersen E. B. (2003). Urinary aquaporin-2 in healthy humans and patients with liver cirrhosis and chronic heart failure during baseline conditions and after acute water load. *Kidney International*.

[21] Hamrahian S. M., Falkner B. (2017). Hypertension in chronic kidney disease. *Advances in Experimental Medicine and Biology Hypertension: from Basic Research to Clinical Practice*.

